# Quality of life in people with ongoing symptoms of coeliac disease despite adherence to a strict gluten-free diet

**DOI:** 10.1038/s41598-020-58236-8

**Published:** 2020-01-24

**Authors:** Joanna E. Harnett, Stephen P. Myers

**Affiliations:** 1 School of Pharmacy, Faculty of Medicine and Health, The University of Sydney, Sydney, Australia; 2 NatMed-Research, Division of Research, Southern Cross University, Lismore, Australia

**Keywords:** Health care, Quality of life

## Abstract

People with Coeliac disease who suffer persistent symptoms despite adherence to a gluten-free diet are at a greater risk of a reduced health related quality of life. The purpose of this paper is to report the quality of life experienced by this specific group of patients in Australia. A Coeliac Disease Specific Questionnaire (CDQ) was administered to 45 people who were enrolled in a clinical trial and reported persistent symptoms of Coeliac disease despite adherence to a strict gluten free diet. The clinical trial was based in New South Wales, Australia. The instrument used was a subscale and total scores of a CDQ measuring health related quality of life. At baseline the overall mean CDQ score was 147 ± 3.31 (optimum 196) consisting of 4 subscales; gastrointestinal 33 ± 0.88, emotional 32.9 ± 0.99, worries 39.8 ± 0.79 and social 41 ± 6.12 each with a potential score of 49. The health related quality of life of people reporting persistent symptoms of Coeliac disease despite adherence to a gluten free diet is sub-optimal with concerningly low scores for emotional quality.

## Introduction

Coeliac disease (CoeD) is a chronic inflammatory autoimmune disorder of the small intestinal mucosa, triggered by the ingestion of gluten proteins in a genetically predisposed population^1^. The treatment of CoeD requires the patient strictly adheres to a gluten free diet. Despite strict adherence to a gluten free diet (GFD) a substantial number of patients report only partial symptom resolution^2^. A nationwide study in Finland of 596 adults with CoeD demonstrated that up to 25% suffered persistent symptoms^3^. The most common cause of persistent symptoms is inadvertent ingestion of gluten containing foods^4^. Other possible causes include small intestinal bacterial overgrowth^5^, transient lactose intolerance^4^, pancreatic insufficiency^4^, poorly absorbed short chain carbohydrates^6^ and gastrointestinal comorbidities^4^.

The increased risk of reduced quality of life in individuals with CoeD who suffer persistent symptoms despite strict adherence to a gluten-free diet led to the incorporation of the Coeliac Disease Specific Questionnaire (CDQ) within a clinical trial looking at the effect of a probiotic supplement in alleviating the persistent symptoms. The results of the effects of probiotic supplement taken over 12 weeks on the primary outcome (faecal microbiota) or secondary outcomes (symptom scores and quality of life) has been reported elsewhere^7^. Briefly, the study found no significant difference in either primary or secondary outcomes between the probiotic supplement and placebo. However, independent of the findings of the clinical trial, the baseline data collected from the CDQ provided a stand-alone cross-sectional snapshot of the quality of life (QoL) of this population that warrants wider review and discussion.

To date, the QoL in this population has lacked detail and as such been poorly reported in the literature^8^. The authors of a recent systematic review and metanalysis investigating the influence of dietary adherence on normalization of health-related quality of life in people living coeliac disease stated they were unable to report on the effect of persistent symptoms despite dietary adherence on the health related quality of life due to a lack of data on this topic^8^.

Therefore, the purpose of this paper is to report on the quality of life in this specific group of patients, with CoeD who have persistent symptoms despite adherence to a gluten free diet. The results of the clinical trial have been reported elsewhere^7^.

## Methods

### Study design

A cross-sectional Coeliac Disease Questionnaire (CDQ) questionnaire was administered to a sample of convenience that involved 45 people enrolled in a clinical trial^7^.

The clinical trial that incorporated the CDQ was approved by the Human Research Ethics Committee of Southern Cross University (ethics approval number ECN-10-008). The research was conducted in compliance with Good Clinical Practice (GCP) and in accordance with the guidelines of the Australian National Health and Medical Research Council and the Declaration of Helsinki (as revised in 2004). The trial was registered with the Australian and New Zealand Clinical Trials Register (ACTRN12610000630011).

### Study population

People living with CoeD who continued to experience persistent gastrointestinal symptoms despite self-reported compliance to a strict GFD for the previous twelve months were invited via the New South Wales Coeliac Association email data base to express their interest in a clinical trial. All participants received a participant information statement outlining the study and signed an informed consent form agreeing to participate when they attended an initial screening interview. Inclusion criteria were: a) CoeD had been confirmed by a small bowel biopsy greater than twelve months prior to enrolling in the study; b) between 18 and 70 years of age b) and c) were taking a GFD for a minimum of twelve months. It was proposed post-hoc that evidence of partial or complete villous architecture repair and/or normalisation of tissue transglutaminase (tTg) antibody levels and/or endomysial antibody levels as an additional inclusion factor would have provided a more homogenous study population. Therefore, sensitivity analysis was undertaken on the inclusion criteria a priori and with the effect of this post-hoc criteria addition.

Individuals were excluded if they were: a) less than 18 years of age; b) pregnant; c) consuming gluten in their diet; d) diagnosed with CoeD within the previous 12 months; e) diagnosed with a major gastrointestinal condition (e.g. inflammatory bowel disease); f) short bowel syndrome; g) recent bowel or oral surgery; h) HIV-positive, cancer, current alcohol and/or illicit drug dependence i) use of non-steroidal anti-inflammatory drugs, antibiotics or steroids in the four weeks before the start of the trial; j) clinically concerning abnormalities in serum urea, electrolytes, liver function or creatinine values; k) unwilling to adhere to the clinical trial component of the study protocol or that in the views the investigators could compromise the study.

### Outcome measures

The scores obtained from a validated health related quality of life instrument the Coeliac Disease Questionnaire (CDQ)^9^.

### CoeD specific questionnaire

The CDQ, a survey instrument employed in this study was developed using a methodological framework following pre-defined criteria of testing theory and standards for validating health measures^9^. The German version of the CDQ was originally adapted to the English language by a forward-backward translation conducted by 1 medical and 1 non-medical professional bilingual translator who evaluated the CDQ for readability and ease of completion^9^. The CDQ has since been validated in Turkish^10^, French^11^, and Italian^12^. Permission was obtained for use of the CDQ in this present  study that involved an English speaking Australian population^9^. Two questions from the ‘worry’ scale (Q24 and Q25) were reworded to ensure relevance to the cultural context and healthcare system that the Australian participants were living in.

The CDQ scores were calculated using measures obtained from the participants rating their own symptoms and QOL. Participants were asked to score the twenty-eight questions on a likert scale of 1–7 (rating the frequency and or severity of symptoms i.e. 1 = all the time, 7 = none of the time) at baseline and at weeks four, eight, twelve and 16 of the study period. The questions were categorised into four sub-scales (emotion, worry, social and gastrointestinal symptoms). Each of the four sub-scales’ scores were then calculated to provide a sub-scale score for the individual areas of interest. The sub-scale scores range between 0–49 in each sub-scale. The total score of all four sub-scales was calculated at baseline and again at week twelve. The total score ranges between 0–196. High scores are indicative of a high health-related quality of life, low scores indicate a reduced health-related quality of life.

In addition, the clinical interview and assessment conducted at baseline included questions that would identify ‘red flag signs and/or symptoms’ for more serious gastrointestinal pathology.

### Adherence to a gluten free diet (GFD): the three-day diet diary

Compliance to the recommended treatment of a GFD was assessed by reviewing a three-day diet diary at baseline, week six and week twelve. Where the regular inadvertent or deliberate ingestion of gluten containing foods was reported at baseline, participants were excluded from the study. If gluten was reported at weeks 6 and 12, it was considered a confounding factor in the statistical analysis.

### Statistical methods

The statistical packages used were SPSS PASW®Statistics GradPack 18 and version 20 SPSS. Descriptive statistics were conducted to describe the characteristics of the sample population and enter scores obtained from the CDQ.

## Results

Forty five people living with CoeD and reporting ongoing symptoms completed a CDQ at baseline.

### Demographics

As presented in Table 1, Forty five participants enrolled (8 males and 37 females) with a mean age at baseline of 47.5 years of age (SD + 12.87). All participants resided in New South Wales (n = 43) or the Australian Capital Territory (n = 2).

**Table 1:** Demographics and characteristics of participants reporting persistent symptoms of Coeliac disease.

Demographic Data n = 45	n = (%)
Females	36 (80)
Males	9 (20)
Age mean (±SD)	47.5 (±12.87)*
No family history of Coeliac disease	30 (67)
Family history of Coeliac disease	15 (33)
No improvement of villous architecture after >12 months of gluten free diet	2 (4)
Partial improvement of villous architecture after >12 months of gluten free diet	21 (47)
Full villous architecture restoration after >12 months of gluten free diet	11 (24)
No follow-up biopsy conducted	10 (22)
Known food intolerance other than gluten	23 (51)

### Clinical characteristics of participants

All 45 participants met the diagnostic criteria for CoeD as outlined by The Australian Coeliac Association. Participants reported only partial symptom improvement in response to the GFD and were bothered with residual gastrointestinal symptoms and fatigue despite reporting adherence to a GFD for >12 months. The severity of symptoms, as rated by the CDQ were mild to moderate at baseline^9^. The gastrointestinal symptoms measured included incomplete defecation, bloating and flatulence, abdominal discomfort and cramping, urgency to defecate, loose stools, eructations and nausea.

All participants reported the normalisation of coeliac serology, with the exception of two participants. Ten participants who reported normalisation of serology after 12 months of adherence to a GFD had not had a follow-up biopsy (see Table 1). No participant reported any ‘red flag’ signs or symptoms such as unexplained weight loss or fever, severe and persistent symptoms, blood in the stool or black stools. All participants were under the care of a gastroenterologist and/or their general practitioner.

Table 1 presents the demographic information and characteristics of the respondents. Participants reported either persistent villous atrophy (n = 2), partial villous architecture repair plus normalisation of coeliac serology (n = 20) or full villous architecture repair plus normalisation of serology (n = 10) or normalisation of serology (n = 10) prior to the study commencing. Sensitivity analysis with the Post hoc removal of the two participants with persistent villous atrophy did not alter any of the results described.

### Results of the coeliac disease questionnaire

The mean scores including lower and upper confidence levels for total quality of life and each of the four subscales gastrointestinal, worries, social and emotional quality of life are reported in Table 2. The lowest scores reported were for emotional wellbeing, followed by gastrointestinal, worries and social subscales.

**Table 2:** The effect of time for subscales emotion, worries, gastrointestinal, social and total Coeliac Disease Questionnaire Scores*. (n = 45).

CDQ Scale	Mean Scores	SD	Lower CI (95%)	Upper CI (95%)
Emotion	32.9	0.99	30.9	34.8
Gastrointestinal	33.0	0.88	31.2	34.8
Worries	39.8	0.79	37.2	40.0
Social	41.9	6.5	19.0	49.0
Total Scores for CDQ	147	3.31	141	154

*The minimum score for each subscale was 7 and the maximum score was 49. The minimum total score was 49 and maximum 196.

## Discussion

The assessment of health related quality of life (HRQOL) is an important outcome measure in clinical studies in gastroenterology^9^. Independent of the findings of the randomised clinical trial, this paper reports on the quality of life of a cohort of adults with CoeD who have persistent symptoms. At baseline, these individuals demonstrated an overall mean score on the CDQ of 147 ± 3.31 of a potential 196 points. This observation is consistent with the observation of decreased quality of life in this cohort (6) and in CoeD in general^13^. Importantly, the emotional well-being subscale mean scores at baseline 32.9 ± 0.99 was substantially less than the optimum score of 49 and is concerning.

Anxiety and depression along with fatigue are the most common extra intestinal complaints in people living with CoeD, especially those who have not yet commenced dietary treatment^13^. Anxiety symptoms measured using the Hospital Anxiety and Depression Scale (HADS) that were consistent with probable anxiety disorder were demonstrated in 16.8% in adults with CoeD on a gluten-free diet (n = 441) compared to 5% in a representative sample of the general German population^14^. Interestingly, this group did not demonstrate any increase in probable depressive disorder between those with CoeD and the general population^14^. This last finding is consistent with a recent US national survey that found no increased incidence of depression in participants with a CoeD diagnosis (3.9%; n = 106) compared to controls (8.9%: n = 22,955) as part of the National Health and Nutrition Examination Survey^15^. Paarahti *et al*. found that the prevalence of depression diagnosis in a larger cohort of adults with coeliac disease (n = 596) was 2%^3^. As a component of the same epidemiological study they demonstrated that adults with CoeD who had persistent symptoms had a much greater risk of reduced health quality of life, however, they did not report on the mental health status of this cohort specifically. We would hypothesise that this group, those with persistent symptoms despite a gluten-free diet, is likely to be over represented in adults with CoeD presenting with depression and we recommend that this be further investigated.

Based on the result of the low emotional well-being score found we would advocate that all CoeD patients presenting with persistent symptoms despite adherence to a gluten-free diet need to be screened for emotional well-being and mental health disorders notably anxiety and depression in addition to other medical investigations required. Additionally, regardless of any specific diagnosis, those individuals with low emotional well-being need to be provided with appropriate support and resources to enhance their emotional quality of life.

A potential limitation of this study relates to the representation of the sample. It has been estimated that up to 25% of individuals with CoeD have persistent symptoms despite reporting adherence to a gluten free diet. The association between persistent symptoms and QoL presented in this study was a secondary outcome of a clinical trial. The study was only powered towards the primary outcome measure of the clinical trial. Future studies should consider ensuring a larger representative sample in their study design. In addition, the act of enrolling in a clinical trial may be a form of filtering that inadvertently created a selection bias that would differ should we have recruited participants to complete the CDQ in a survey design outside the context of a clinical trial.

Despite this limitation, the sub optimal scores especially in gastrointestinal symptoms and low emotional quality of life are concerning. In addition to providing the expertise and support required for patients to successfully adopt and adhere to a GFD health care providers should be aware of the higher prevalence of mental health issues in this population. As such, the mental health and wellbeing of individuals who report experiencing persistent symptoms of CoeD despite adherence to a GFD should be considered as part of a thorough clinical assessment.

## References

[1] Lebwohl B, Sanders DS, Green PHR (2018). Coeliac disease. The Lancet.

[2] Stasi E (2016). Frequency and cause of persistent symptoms in celiac disease patients on a long-term gluten-free diet. Journal of clinical gastroenterology.

[3] Paarlahti P (2013). Predictors of persistent symptoms and reduced quality of life in treated coeliac disease patients: a large cross-sectional study. BMC Gastroenterology.

[4] Green PHR, Cellier C (2007). Celiac disease. New England Journal of Medicine.

[5] Tursi A, Brandimarte G, Giorgetti G (2003). High prevalence of small intestinal bacterial overgrowth in celiac patients with persistence of gastrointestinal symptoms after gluten withdrawal. The American Journal Of Gastroenterology.

[6] Gibson PR, Shepherd SJ (2010). Evidence-based dietary management of functional gastrointestinal symptoms: The FODMAP approach. Journal Of Gastroenterology And Hepatology.

[7] Harnett, J., Myers, S. P. & Rolfe, M. Probiotics and the Microbiome in Celiac Disease: A Randomised Controlled Trial. *Evidence-Based Complementary and Alternative Medicine*, 16 (2016). 10.1155/2016/9048574 PMC4972910 27525027

[8] Burger JPW (2017). Systematic review with meta-analysis: Dietary adherence influences normalization of health-related quality of life in coeliac disease. Clinical Nutrition.

[9] Häuser W, Gold J, Stallmach A, Caspary WF, Stein J (2007). Development and validation of the Celiac Disease Questionnaire (CDQ), a disease-specific health-related quality of life measure for adult patients with celiac disease. Journal of clinical gastroenterology.

[10] Aksan A, Mercanlıgil SM, Häuser W, Karaismailoğlu E (2015). Validation of the Turkish version of the Celiac Disease Questionnaire (CDQ). Health and quality of life outcomes.

[11] Pouchot J (2014). Validation of a French version of the quality of life “Celiac Disease Questionnaire”. PloS one.

[12] Zingone F (2013). The Italian translation of the celiac disease-specific quality of life scale in celiac patients on gluten free diet. Digestive and Liver Disease.

[13] Cossu G (2017). Coeliac disease and psychiatric comorbidity: epidemiology, pathophysiological mechanisms, quality-of-life, and gluten-free diet effects. International Review of Psychiatry.

[14] Häuser W, Janke K-H, Klump B, Gregor M, Hinz A (2010). Anxiety and depression in adult patients with celiac disease on a gluten-free diet. World journal of gastroenterology: WJG.

[15] Zylberberg HM, Demmer RT, Murray JA, Green PH, Lebwohl B (2017). Depression and insomnia among individuals with celiac disease or on a gluten-free diet in the USA: results from a national survey. European Journal of Gastroenterology & Hepatology.

